# Epidemiological factors affecting outpatient department service utilization and hospitalization in patients with diabetes: A time-series analysis from an Ethiopian hospital between 2018 and 2021

**DOI:** 10.7189/jogh.12.04087

**Published:** 2022-10-23

**Authors:** Roberto Benoni, Anna Sartorello, Monica Uliana, Hiwot Solomon, Alessia Bertolino, Andrea Pedot, Ademe Tsegaye, Berhanu Gulo, Fabio Manenti, Giacomo Andreani

**Affiliations:** 1 Department of Diagnostics and Public Health, University of Verona, Verona, Italy; 2 Doctors with Africa CUAMM, Padova, Italy; 3 Department of Internal Medicine IV, AOU Pisana, Pisa, Italy; 4 Disease Prevention and Control Directorate, Federal Ministry of Health, Addis Ababa, Ethiopia; 5 Division of Paediatric Surgery, Department of 'Salute della Donna e del Bambino', University of Padova, Padova, Italy; 6 School of Medicine and Surgery, Dept. of Medicine, University of Padova; 7 Doctors with Africa CUAMM, Addis Ababa, Ethiopia; 8 Department of Clinical and Biological Sciences, University of Turin, San Luigi Gonzaga Hospital, Orbassano, Turin, Italy; 9 Emergency Department and High-dependency Unit, Cardinal Massaia Hospital, Asti, Italy

## Abstract

**Background:**

The burden of diabetes-related deaths reached two million in 2019 globally. Accessibility to health care services and adherence to follow-up and therapy are key to improving outcomes for diabetic patients. We aimed to assess outpatient department (OPD) service utilization and diabetes-related hospitalizations over a period of 44 months.

**Methods:**

A retrospective cohort study was conducted on OPD visits and hospitalizations recorded between January 1, 2018, and August 31, 2021, at the St Luke Catholic Hospital (Ethiopia). All diabetic patients were included in the analysis. A linear regression model was used for univariate analysis of OPD visits and hospitalizations and their association with potential predictors. The autoregressive integrated moving average (ARIMA) method was applied to both the time series of OPD visits and hospitalizations. Potential predictors were sociodemographic factors, COVID-19 cases, mean monthly temperature and precipitations.

**Results:**

In the time series analysis, OPD visits increased over time (*P* < 0.01) while hospitalizations were stable. The time series model was ARIMA (0,1,1) for OPD visits and ARIMA (0,0,0) for hospitalizations. There were 1685 diabetes OPD patients (F = 732, 43%). Females had an average of 16% fewer OPD accesses per month (*P* < 0.01) and a lower number of hospitalizations per month (*P* = 0.03). There were 801 patients missing follow-up (48%). The time between follow-up increased with age (*P* < 0.01). OPD visits decreased differently by geographic area as COVID-19 cases increased (*P* < 0.01). There were 57 fewer forecast OPD visits per month on average using COVID-19 cases as ARIMA regressor. The odds ratio (OR) of new diagnosis at hospitalization was lower in patients with type 2 diabetes (OR = 0.26, 95% CI = 0.14-0.49, *P* = 0.02).

**Conclusions:**

Despite an increase in OPD visits for diabetic patients over the study period, the number of losses at follow-up and diagnoses at hospitalization remains high. Female sex, older age, and COVID-19 were associated with impaired OPD service accessibility. Primary health care should be implemented to achieve better health coverage and improve diabetes management.

Non-communicable diseases (NCDs) are the leading cause of death globally. The four major NCDs are cancer, chronic vascular diseases, diabetes mellitus, and chronic respiratory diseases [1]. The global share of NCD deaths among all deaths grew from 60.8% in 2000 to 73.6% in 2019, reaching 33.2 million. Most NCDs related deaths are concentrated in low- and middle-income countries (LMICs), where 10.5 million deaths are accountable to these four diseases in 2019 only. Currently, Ethiopia has been challenged by the burden of NCDs which are estimated to be liable for 39% of all deaths [2].

Among NCDs, diabetes mellitus is an important cause of morbidity, mortality, and a burden for health systems around the world. Global diabetes deaths grew by 72% since 2000, reaching almost two million in 2019. The International Diabetes Federation estimated that 463 million people had diabetes in 2019, and this number is projected to reach 578 million by 2030, and 700 million by 2045 [3]. The greatest increase in incidence and the highest proportion of undiagnosed diabetes mellitus was observed in the African continent (59,7%) [4].

Management and clinical outcome of diabetic patients are linked to the accessibility of health care facilities, availability of screening, and follow-up visits [5]. In LMICs, barriers to health care access are more numerous and more extreme than in high-income countries (HICs). Gender is the most common demographic barrier, followed by cultural factors. Financial factors combine with geographical factors; indirect costs due to long travels to health care facilities add to direct prices, often excessively high [6].

Health care access has changed during the COVID-19 pandemic: all over the world, facility utilization decreased by one-third. Service reduction was prominent in LMICs [7]. The impact of COVID-19 on health care systems was linked to insufficient staff availability, fear of becoming infected while accessing hospitals, and lockdown policies [8]. Concerning NCDs, half of the countries worldwide reported disruptions to diabetes mellitus management services [9].

Although adherence to visits is critical in chronic diseases, such as diabetes, few studies have explored factors associated with health care services utilization and accessibility in LMICs. The primary objective of this study was to assess the impact of sociodemographic and environmental factors of patients with diabetes on service utilization (in terms of access per month, losses at follow-up, and readmissions). The secondary objective was to explore the impact of COVID-19 on access to the outpatient department (OPD) service and to hospitalizations. The main hypothesis was that access to health care services was impaired by factors such as female sex and older age and that access decreased during the rainy season.

## METHODS

### Study design

A retrospective cohort study design was used to analyse the role of sociodemographic and environmental factors on health service utilization by patients with diabetes.

### Setting

St Luke Catholic Hospital (SLCH) is based in South-West Shewa Zone (SWSZ), Oromia region (Ethiopia). It has an estimated population of 1 311 406 inhabitants [10]. The SWSZ is divided into twelve districts called “woreda”; these are the third level of the administrative division. SLCH is a general hospital located in Wolisso Town, 114 km from the capital Addis Ababa. Its catchment area includes the woreda of Ameya, Wenchi, Wolisso rural, Wolisso town, Becho, and Goro representing the reference hospital for 743 797 individuals. The hospital has 200 beds with an annual average bed occupation rate of 94.1%.

### Data collection

Data on the number of outpatient department (OPD) visits and hospitalizations, age, sex, residence at woreda level, date of hospital access, type of diagnosis, and data on the outcome (only for hospitalized patients) were obtained from the registration software of the SLCH, Open Hospital, from January 1, 2018, to August 31, 2021.

To explore potential determinants in the monthly number of OPD visits and hospitalizations during the study period, the number of monthly newly registered COVID-19 cases in Ethiopia and the mean monthly values of precipitation millimetres and temperature (°C) estimated in the Oromia region between 1991 and 2020 were collected [11,12].

### Operational Definitions

According to Ethiopian guidelines, the following criteria were adopted by hospital doctors for the diagnosis of diabetes [13]:

1. A diabetes case was diagnosed if the patient had a fasting blood sugar (FBS) ≥126 mg/dL (at least two tests needed), or random blood sugar (RBS) ≥200 mg/dL plus classic symptoms of hyperglycaemia (i.e., fatigue, polyuria, polydipsia, blurred vision, recurrent skin infections), hyperglycaemic crisis, or symptoms related to chronic complications.
2. When a patient was diagnosed with diabetes and presented with classic symptoms such as excessive urination or thirst, unexplained weight loss, or if the patient had suggestive sociodemographic characteristics (i.e., young age), he/she was diagnosed with type 1 diabetes.
3. When a patient met the criteria for the diabetes diagnosis and presented with other symptoms such as overweight or obese, chronic diabetic complication (i.e., numbness or pain over the lower limbs, visual impairment, foot abnormalities (such as ulcer, ischaemia, deformity), body swelling), had a family history of diabetes, dyslipidaemia, or was aged 45 years old or more, he/she was diagnosed with type 2 diabetes.

### Study endpoints

The primary endpoints were the number of OPD service accesses and the number of hospitalizations per month in patients with diabetes. The secondary endpoint was the frequency of patients with diabetes not attending a follow-up visit and requiring hospital readmission.

### Statistical analysis

#### Descriptive analysis

Categorical variables were presented as frequencies and proportions, while continuous variables were presented as means and standard deviations (SDs) or medians and interquartile ranges (IQRs). Sample distribution was tested via χ2 and Fisher exact test for categorical variables or Mann-Whitney-U non-parametric or ANOVA test for continuous variables, as appropriate. The Shapiro-Wilks test was used to test the normality of the distribution of the included variables. Logistic regression models were fitted for multivariable analyses, where follow-up visit (yes/no), first diagnosis during hospitalization (yes/no), readmission (yes/no), and readmission within 30 days (yes/no) were the response variables, and type of diabetes, age and sex were potential determinants. Results were presented as odds ratio (OR) with 0.95 confidential intervals (CIs).

#### Time series analysis

Univariate analysis of monthly OPD visits and hospitalizations for diabetes and their association with potential predictors was carried out using a general linear model. Independent variables were the time (in months), sociodemographic factors, precipitations, temperature, and the number of COVID-19 cases newly registered in Ethiopia.

The time series analysis was applied to monthly OPD visits and hospitalizations for diabetes. To identify the trend of the time series, LOcally Weighted Scatterplot Smoothing (LOWESS) function was used to test different smoother spans (f = 2/3, f = ⅓, and f = 0.1). The trend was then subtracted from the time series to isolate the random error (remainder component).

AutoRegressive Integrated Moving Average (ARIMA) model was applied to identify significant predictors as well as to forecast the number of OPD visits and hospitalizations [14].

The p, d, and q of the model were chosen by combining unit root tests, the minimization of the Akaike information criterion (AIC), and Maximum Likelihood Estimation (MLE). To choose the best ARIMA model, an algorithm was applied using a stepwise search to traverse the model space selecting the best model with the smallest AIC. The KPSS test was used to determine the number of differences (d) in the Hyndman-Khandakar algorithm for automatic ARIMA modelling [15,16].

The ARIMA model was applied to the time series of the monthly number of OPD visits and hospitalizations. First, to forecast the number of monthly OPD visits and hospitalizations, an ARIMA model was fitted with the previously registered values. The number of periods (h) was set at six months. Then, the same analysis was repeated with the monthly number of Ethiopian newly registered cases of COVID-19 as a regressor in the ARIMA models to estimate the impact of the COVID-19 pandemic on hospital accessibility. The residuals from the ARIMA models have been tested for no autocorrelation through the Ljung-Box Tests [17].

All analyses were performed using R software (version 4.1.1). A *P*-value <0.05 was considered signiﬁcant.

### Ethical approval

The inclusion of a patient in this protocol did not require any additional exams besides those normally needed for clinical routine. The research was performed following the ethical standards of the 1964 Declaration of Helsinki and was approved by the Ethical Committee of St Luke Catholic Hospital on May 26, 2021 (protocol number 665/41).

## RESULTS

### Sample characteristics

In the study period, 93 301 patients from the SWSZ were assisted at the OPD of the SLCH and 1685 (1.8%) for diabetes. Sex distribution was 732 females (43.4%) and 953 males (56.6%) (**Table 1**). Females had a mean age of 38.8 years (SD = 19.3) and were younger compared to males (mean = 42.0 years, SD = 19.8, *P* < 0.01). Of all the patients with diabetes, 467 (28.2%) were diabetes mellitus type 1 (DM1) with a mean age of 18.7 years (SD = 14.3) and 1218 (71.8%) were diabetes mellitus type 2 (DM2) with a mean age of 49.0 years (SD = 14.0). There were no differences in sex distribution between types of diabetes (*P* = 0.14) (**Table 1**). The first visit at SLCH resulted in a first diagnosis for 1522 patients (90.3%), and 84 (5.5%) of these occurred because of hospitalization.

**Table 1:** Characteristics of diabetic patients attending OPD services and admitted to medical and paediatric wards of the St. Luke Catholic Hospital between January 1, 2018, and August 31, 2021, distinguished by diabetes type

	Type 1 (OPD = 467, H = 188)	Type 2 (OPD = 1218, H = 220)	*P-*value*	Overall (OPD = 1685, H = 48)
**Diabetes OPD visits**				
**Sex**				
Female	245 (52.5%)	487 (40.0%)	0.02	732 (43.4%)
Male	268 (57.4%)	685 (56.2%)		953 (56.6%)
**Age (years – mean (SD))**	18.7 (14.3)	49.0 (14.1)	<0.01	40.6 (19.6)
**At least 1 follow-up visit**				
Yes	192 (41.1%)	692 (56.8%)	0.10	884 (52.5%)
No	420 (81.9%)	893 (76.2%)		801 (47.5%)
**First follow-up <30 days**				
Yes	192 (41,1%)	692 (56,8%)	0.51	372 (22.1%)
No	275 (58,9%)	526 (43,2%)		1313 (77.9%)
**Time between follow-up (days – mean (SD))**	60.3 (87.2)	64.7 (84.4)	0.25	63.8 (84.9)
**First diagnosis during hospitalization** **(N/A = 163)**				
Yes	44 (10.1%)	41 (3.7%)	0.02	84 (5.5%)
No	384 (89.9%)	1054 (96.3%)		1437 (94.5%)
**Diabetes hospital admissions**				
**Sex**				
Female	88 (46.8%)	89 (40.5%)	0.23	177 (43.4%)
Male	100 (53.2%)	131 (59.5%)		231 (56.6%)
**Age (years – mean (SD))**	17.8 (12.1)	49.7 (13.0)	<0.01	35.0 (20.3)
**Length of stay (days – mean (SD))**	6.9 (5.9)	5.9 (5.8)	0.37	6.4 (5.9)
**Readmission**				
Yes	31 (16.5%)	21 (9.5%)	0.30	52 (12.7%)
No	157 (83.5%)	199 (90.5%)		356 (87.3%)
**Readmission within 30 days**				
Yes	6 (3.2%)	4 (1.8%)	0.48	10 (2.5%)
No	182 (96.8%)	216 (98.2%)		398 (97.5%)
**Outcome**				
Death	5 (2.7%)	7 (3.2%)	0.75	12 (2.9%)
Alive	183 (97.3%)	213 (96.8%)		396 (97.1%)

OPD – outpatient department services, H – hospitalizations, SD – standard deviation

*Fisher exact test, χ^2^ test, analysis of variance (ANOVA), logistic regression.

The median number of visits per patient was 2 (IQR = 1-6); 801 (47.5%) patients attended only one OPD visit with no further follow-up. Considering patients (n = 884, 52.5%) with at least one follow-up visit during the study period, the median number of visits per person increased to 6 (IQR = 3-11). The mean time between follow-up visits was 63.8 days (SD = 84.9). The odds ratio of having at least one follow-up visit increased with age (OR = 1.84; 95% CI = 0.098-0.223, *P* = 0.03). There were no differences in the time between follow-ups based on patient sex (*P* = 0.98) or type of diabetes (*P* = 0.25); the time between visits was longer as the age of the patients increased (*P* < 0.01).

In the study period, 8619 patients from the SWSZ were admitted to the medical and paediatric wards of the SLCH and 408 (4.7%) had diabetes (**Table 1**). Females had a mean age at admission of 31.4 years (SD = 19.8) and were younger than males (mean = 37.7 years, SD = 20.3; *P* < 0.01).

There were no differences in mean length of stay based on sex (*P* = 0.17) or diabetes type (*P* = 0.37), while it decreased as age increased (*P* = 0.01). There were 52 (12.7%) patients with more than one hospitalization with a median readmission number of 1.0 (IQR = 1.0-3.0). The median readmission time was 132.2 days (IQR = 50.9-279.8). There were no differences in the odds ratio of readmission based on sex (*P* = 0.86), age (*P* = 0.99), or diabetes type (*P* = 0.11). The odds ratio of receiving a new diagnosis at hospitalization was lower in DM2 (OR = 0.26; 95% CI = 0.14-0.49, *P* = 0.02) with no differences based on sex (*P* = 0.45) or age (*P* = 0.19).

### Time series analysis

**Figure 1:**
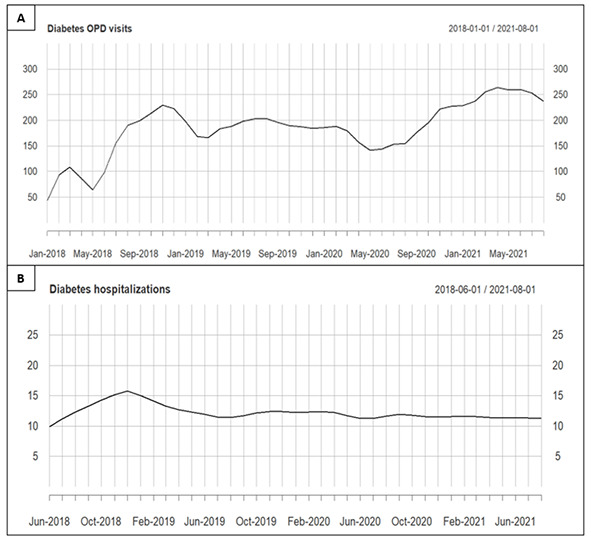
Time trend of diabetes OPD visits (panel A) and hospitalizations (panel B) between January 2018 and August 2021.

The total number of OPD visits per month between January 1, 2018, and August 31, 2021, was considered together with the number of COVID-19 cases newly registered in Ethiopia (Table S1 in the **Online Supplementary Document**). The mean rain precipitation millimetres and temperature per month are shown in Table S3 in the **Online Supplementary Document**. Considering the time series of the number of OPD visits, the lowess function with f = 0.1 was found to better fit the trend (Figure S1 in the **Online Supplementary Document**). The decomposition of the time series is shown in Figure S2 in the **Online Supplementary Document**. The trend of diabetes OPD visits is shown in **Figure 1** (panel A).

**Figure 2:**
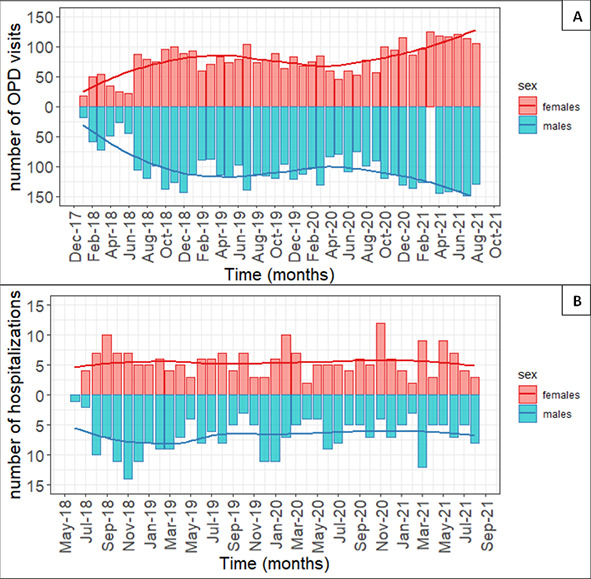
Divergent bar chart and time trend of the number of outpatient department (OPD) visits (panel A) and of hospitalizations (panel B) from 01/01/2018 and 31/08/201) distinguished by sex (females = red coloured and upper part of the graphs; males = blue coloured and lower part of the graphs).

The number of OPD visits for diabetes increased by 2.8 (standard error (SE) = 0.5) over the months (*P* < 0.01), while neither the mean monthly temperature (*P* = 0.21) nor precipitation (*P* = 0.99) was associated with OPD attendance for diabetes. Males had an average of 16% more OPD visits per month than females (β = 28.4, SE = 4.9; *P* < 0.01) (**Figure 2**, panel A).

The best-fitting model for OPD visits time series was ARIMA (0,1,1), which is a non-seasonal and non-stationary moving average model. The residuals from the ARIMA model had no autocorrelation (Q = 6.7, degrees of freedom (df) = 8; *P* = 0.57, Figure S3 in the **Online Supplementary Document**). The mean forecast number of the OPD visits for the following six months (September 2021 to February 2022) was 243.7 (95% CI = 135.6-351.80) (**Table 2**).

**Table 2:** Forecast number of diabetes OPD visits fitted without (ARIMA 0,1,1) and with (ARIMA 1,0,0) monthly number of newly registered COVID-19 cases as a regressor

	ARIMA (0,1,1)		ARIMA (1,0,0)	
	Forecast	95% CI	Forecast	95% CI
September 2021	243.7	166.2-321.2	199.6	121.2-278.0
October 2021	243.7	151.9-335.5	192.3	95.1-289.5
November 2021	243.7	139.5-347.9	186.9	81.0-292.9
December 2021	243.7	128.5-358.9	183.1	78.8-293.4
January 2022	243.7	118.4-369.0	180.2	67.6-292.8
February 2022	243.7	109.1-378.4	178.1	64.3-291.9

ARIMA – autoregressive integrated moving average, CI – confidence interval

Comparing the monthly number of OPD visits during the first months of the pandemic (March 202 to August 2020) with the same period in 2019, there was a 28% reduction in overall diabetes visits (Table S1 in the **Online Supplementary Document**). The best-fitting model for OPD visits time series, using the monthly number of newly registered COVID-19 cases as a regressor, was ARIMA (1,0,0), which is a non-seasonal, stationary, autoregressive model. The residuals from the ARIMA model had no autocorrelation (Q = 8.8, df = 6; *P* = 0.184, Figure S4 in the **Online Supplementary Document**). The mean forecast number of the OPD visits for the following six months (September 2021 – February 2022), based on the number of monthly newly registered COVID-19 cases, was 186.7 (95 CI% = 84.7-289.8) (**Table 2**).

**Figure 3:**
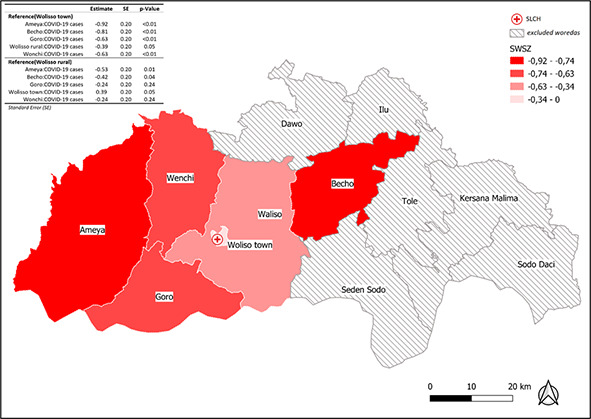
Graphical representation of the coefficients from linear regression model fitted on the number of monthly diabetes outpatient department visits as dependent variable, and months and interaction between the number of monthly newly registered COVID-19 cases and woreda (reference Wolisso town and Wolisso rural) between 01/01/2018 and 31/08/2021. Increasing colour intensity shows lower coefficients (ie, a greater decrease in the number of visits as COVID-19 cases increase). Crossed-out areas show woredas excluded from the analysis because they are out of the catchment area of the St Luke Catholic Hospital (SLCH).

In the multivariable analysis with an interaction term between woreda and the monthly number of newly registered COVID-19 cases, the number of OPD visits for diabetes was lower for all woredas when compared to Wolisso town as the number of COVID-19 cases increased (*P* < 0.01) and was lower for the woreda of Ameya and Becho (*P* = 0.01) when compared to Wolisso rural (*P* = 0.04) (**Figure 3**). There were no differences in the number of monthly diabetes hospitalizations in the same analysis (Wolisso town as reference level: Ameya (*P* = 0.60), Becho (*P* = 0.61), Goro (*P* = 0.42), Wolisso rural (*P* = 0.24), Wonchi (*P* = 0.58)).

Considering the time series of the number of hospitalizations for diabetes, the lowess function with f = 1/3 was found to better fit the trend (Figure S5 in the **Online Supplementary Document**). The decomposition of the time series was shown in the supplementary file (Figure S6 in the **Online Supplementary Document**). The trend of diabetes hospitalizations was shown in **Figure 1** (panel B).

The number of hospitalizations for diabetes remained stable over the months (*P* = 0.54) (Table S3 in the **Online Supplementary Document**). Males had a higher number of hospitalizations per month than females (β = 1.3, SE = 2.3; *P* = 0.03) (**Figure 2**, panel B). Neither the mean monthly temperature (*P* = 0.27) nor precipitation millimetres (*P* = 0.10) were associated with the number of hospitalizations for diabetes. There were no differences in the monthly number of hospitalizations based on the number of COVID-19 cases in Ethiopia (*P* = 0.83).

## DISCUSSION

This study explored the service utilization by patients with diabetes from a rural area of Ethiopia. A gender gap, to the disadvantage of women, was found in OPD service utilization and hospitalizations. Contrary to what was assumed, weather conditions (particularly the rainy season) did not appear to affect access to the hospital.

In the time series analyses, females had 28.4 fewer OPD visits per month than males, showing 16% fewer visits on average. A similar trend was found in the number of hospitalizations per month. Despite this difference in health service utilization, the prevalence of diabetes in Ethiopia was reported to be similar between males and females [18]. Healthcare access may be problematic in African countries, where the gender gap may result in different opportunities and education [19]. The strict connection between health care access inequity and gender is a direct consequence of inequalities suffered by women, such as lower financial resources, lower levels of education, and lower independence within society and family. In many countries, women are discriminated against in terms of access to medical care, leading to higher mortality (the so-called “missing women” concept); this gender gap is particularly evident in Asia and Africa [20].

During the study period, we observed an overall increase in OPD visits, while the number of hospitalizations remained stable. The “Doctors with Africa CUAMM” project in Wolisso existed for several years [21]. This investment in health promotion may have led to higher-than-average health literacy, and consequently, good adherence to screening and follow-up resulting in an increase in OPD access. However, the prevalence of diabetes mellitus is increasing in all countries. Growth in new diagnoses was observed in LMICs and collected data reflect this trend [3].

Many individuals did not return for follow-up after the first diagnosis of diabetes mellitus. The prevalence was higher for DM1 than for DM2 (59% vs 43%). Patient education, perception of risk, and environmental factors such as lack of infrastructure and public transport can influence this loss. Moreover, the time between follow-ups increased with age. This data may suggest fewer health care access possibilities for the elderly and could be explained by difficulties in travelling alone to the health care facilities and arduous support by younger relatives who may need to prioritize providing for their family. Overall, 47.5% of individuals were lost after diagnosis: this data emphasizes the importance of investing in health promotion and infrastructure [22]. This result was also highlighted by the lower 30-day readmission rate in our sample (12.7%) compared with the 20% reported in Western studies [23]. This low readmission rate could be associated with a higher death rate at home due to acute complications, little confidence in the health care system, and difficult transport to the hospital.

The included patients with DM1 had a mean age of 19 years. In sub-Saharan Africa, there was evidence of a spike in diagnoses for DM1 a decade later than in HICs. Prolonged breastfeeding has been proposed to be possibly involved in reducing the incidence and delaying the onset of DM1 [24]. Additionally, diagnosis criteria may differ in LMICs and the lack of clear official guidelines for diabetes screening in resource-limited health systems can lead to delayed diagnosis [25]. This was confirmed by the high number of patients accessing the SLCH and receiving a first diabetes diagnosis and by the higher odds ratio of new diagnosis at hospitalization for patients with DM1. Our results showed a similar epidemiological pattern to those reported in the literature also regarding the peak age of DM2 (40 to 59 years old). Interestingly, 24% of patients with DM2 were found to be in the 20 to 39 years old range. A similar prevalence was estimated in sub-Saharan Africa by the International Diabetes Federation [24]. Considering this last age range group of young adults with a diagnosis of DM2, we must take into account the possibility of a diagnostic misclassification of some of these patients, whose diagnosis could be re-classified in LADA (Latent Autoimmune Diabetes in Adults), if only they were not lost at follow-up.

Then, we analysed the influence of the weather on OPD accesses: even though a reduction of OPD visits was expected during the rainy season, no seasonality was retrieved in the time series analysis. Climate seasonal changes do not seem to be an important factor to discontinue health care access for both OPD visits and hospitalizations. Other studies reported an influence of geographic and environmental factors on health accessibility, but there is a lack of data on the weather and seasonal impact [6].

In March 2020, the first COVID-19 case was detected in Ethiopia. The time-series analysis shows a deflection in OPD visits during the first months of the pandemic (March to August 2020) with a reduction of 28% in OPD visits compared to the same months of 2019. Moreover, in the forecast projection of OPD visits using new COVID-19 cases as a regressor, the number was lower by 57 visits per month on average, compared to the forecast without considering COVID-19 cases. Interestingly, when analysing the interaction between new COVID-19 cases and woredas in terms of distance from SLCH, woredas influenced the decreasing rate of access, showing a higher negative impact on distant woredas (**Figure 3**). All over the world, essential health services were affected by the COVID-19 pandemic. In Ethiopia, most departments decreased their activity, including outpatient visits. COVID-19 had direct and indirect effects on health care accessibility. While patients may have been hesitant to seek treatment because they may have considered facilities as infected or because of their possible doubts about the expertise of health care providers, lockdown, other restrictions, or poor infrastructures may have made reaching hospitals more difficult for them [26].

There are several limitations to this study. First, data was limited to a single centre; however, the study focuses on a rural area often underrepresented in scientific literature. Second, the retrospective study design used available medical records, which may not have always been complete and/or correctly compiled. However, the research covered more than three years, reaching a consistent number of individuals. Lastly, the diagnosis of diabetes mellitus was often based on glucose measurement alone, lacking glycated haemoglobin (HbA1c) or an oral glucose tolerance test. This circumstance could have led to some inaccuracy in the estimated prevalence of diabetes. Moreover, the lack of autoantibodies tests and the high rate of losses at follow-up could have misled the diabetes type categorizations, especially for those with DM1 (ie, patients with LADA).

## CONCLUSIONS

Factors involved in health care access in the context of this analysis suggest that investment in patient empowerment, gender equality, infrastructures, and travel opportunities in rural areas must be encouraged. Previous studies have proposed diabetes mellitus as a potential tracer for evaluating health care systems [27]. Our findings show that public health is still a challenge in LMICs. Although the number of OPD visits increased during the study period, the high rate of loss to follow-up and of newly diagnosed at hospitalization shows the need for interventions to improve patient health literacy and health services at the local level. We need to consider whether the local health care system is adequate before improving screening programs for diabetes and for all NCDs [28]. Limited accessibility and a health system unable to handle large and growing volumes of NCD-related admissions could jeopardize the achievement of the Sustainable Development Goals set by the World Health Organization for the 2030 Agenda for Sustainable Development.

## Additional material:


Online Supplementary Document
