# Supplementary material for: Epidemiological factors affecting outpatient department service utilization and hospitalization in patients with diabetes: A time-series analysis from an Ethiopian hospital between 2018 and 2021
Source: J Glob Health. 2022 Oct 23;12:04087. doi: 10.7189/jogh.12.04087 (PMC9588158; doi:10.7189/jogh.12.04087)
Supplement: Online Supplementary Document [file jogh-12-04087-s001.pdf]

Online Supplementary Document

Table S1 *Monthly number of overall diabetes OPD visits between January 1st, 2018, and August 31st, 2021, and distinguished by sex and diabetes type. Monthly number of newly registered COVID-19 cases and COVID-19 related deaths reported in Ethiopia in the same period.*

Time	Overall	Females	Males	DM1	DM2	New COVID-19 cases	New COVID-19 related deaths
Jan-18	37	18	19	3	34	0	0
Feb-18	109	50	59	12	97	0	0
Mar-18	126	54	72	22	104	0	0
Apr-18	83	34	49	23	60	0	0
May-18	51	25	26	6	45	0	0
Jun-18	66	22	44	6	60	0	0
Jul-18	193	87	106	23	170	0	0
Aug-18	199	79	120	24	175	0	0
Sep-18	173	74	99	21	152	0	0
Oct-18	233	95	138	22	211	0	0
Nov-18	226	99	127	30	196	0	0
Dec-18	232	89	143	43	189	0	0
Jan-19	206	93	113	23	183	0	0
Feb-19	149	60	89	7	142	0	0
Mar-19	158	70	88	16	142	0	0
Apr-19	197	83	114	17	180	0	0
May-19	191	73	118	21	170	0	0
Jun-19	176	79	97	11	165	0	0
Jul-19	243	104	139	21	222	0	0
Aug-19	186	73	113	11	175	0	0
Sep-19	194	78	116	13	181	0	0
Oct-19	208	89	119	17	191	0	0
Nov-19	159	63	96	14	145	0	0
Dec-19	204	83	121	17	187	0	0
Jan-20	181	68	113	11	170	0	0
Feb-20	177	75	102	11	166	0	0
Mar-20	215	84	131	14	201	26	0
Apr-20	142	59	83	9	133	105	3
May-20	125	46	79	9	116	1041	8
Jun-20	167	59	108	12	155	4674	92
Jul-20	128	53	75	8	120	11684	171
Aug-20	176	77	99	15	161	34601	535
Sep-20	146	56	90	12	134	23237	389
Oct-20	220	100	120	14	206	20801	271
Nov-20	208	94	114	14	194	13905	237
Dec-20	246	115	131	16	230	14190	217
Jan-21	221	85	136	18	203	13386	170
Feb-21	223	96	127	12	211	21422	272
Mar-21	276	125	151	23	253	47517	500
Apr-21	261	117	144	27	234	50853	823
May-21	258	116	142	30	228	14099	477
Jun-21	261	120	141	28	233	4633	155
Jul-21	261	113	148	34	227	4191	65
Aug-21	234	105	129	27	207	27769	290

Diabetes Mellitus type 1 (DM1), Diabetes Mellitus type 2 (DM2)

Table S2 *Monthly number of overall diabetes-related hospitalizations between January 1st, 2018, and August 31st, 2021, and distinguished by sex and diabetes type.*

Time	Overall	DM1	DM2	Females	Males
Jun-18	1	0	1	0	1
Jul-18	6	2	4	4	2
Aug-18	17	5	12	7	10
Sep-18	17	9	8	10	7
Oct-18	18	3	15	7	11
Nov-18	21	4	17	7	14
Dec-18	16	4	12	5	11
Jan-19	13	3	10	5	8
Feb-19	15	2	13	6	9
Mar-19	13	3	10	4	9
Apr-19	12	4	8	5	7
May-19	7	4	3	3	4
Jun-19	14	5	9	6	8
Jul-19	12	2	10	6	6
Aug-19	15	5	10	7	8
Sep-19	9	4	5	4	5
Oct-19	10	1	9	7	3
Nov-19	8	1	7	3	5
Dec-19	14	4	10	3	11
Jan-20	17	5	12	6	11
Feb-20	17	2	15	10	7
Mar-20	12	2	10	7	5
Apr-20	6	1	5	2	4
May-20	9	2	7	5	4
Jun-20	14	2	12	5	9
Jul-20	13	4	9	5	8
Aug-20	9	2	7	4	5
Sep-20	11	2	9	6	5
Oct-20	12	7	5	5	7
Nov-20	16	3	13	12	4
Dec-20	13	1	12	6	7
Jan-21	9	8	1	4	5
Feb-21	5	4	1	2	3
Mar-21	21	13	8	9	12
Apr-21	8	2	6	3	5
May-21	14	6	8	9	5
Jun-21	14	4	10	7	7
Jul-21	9	8	1	4	5
Aug-21	11	5	6	3	8

Diabetes Mellitus type 1 (DM1), Diabetes Mellitus type 2 (DM2)

Table S3 *Mean value of estimated precipitations and temperature per month(1991-2020) in the Oromia region – Ethiopia*

Month	Mean precipitations (mm)	Mean temperature (°C)
January	18.7	19.6
February	22.1	20.7
March	61.6	21.6
April	119.7	21.8
May	146.9	21.3
June	104.7	20.7
July	145.0	19.9
August	158.6	16.6
September	129.8	20.2
October	110.0	19.7
November	44.0	19.1
December	17.8	18.9

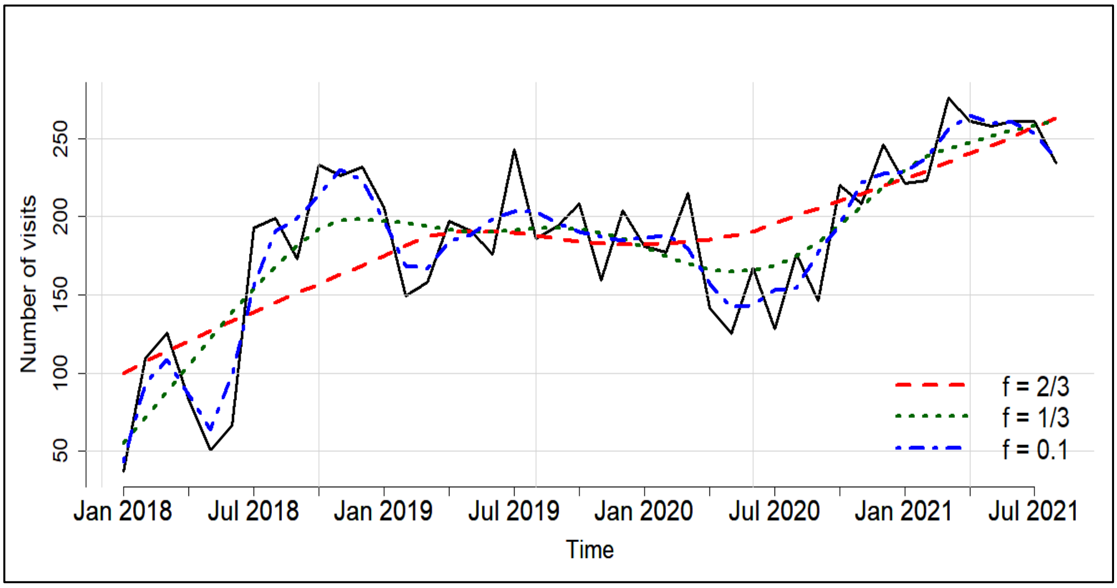

Figure S1 *Local weighted regression smoothing (LOWESS) with different smoothing parameters (f) of diabetes OPD visits time series from January 2018 to August 2021.*

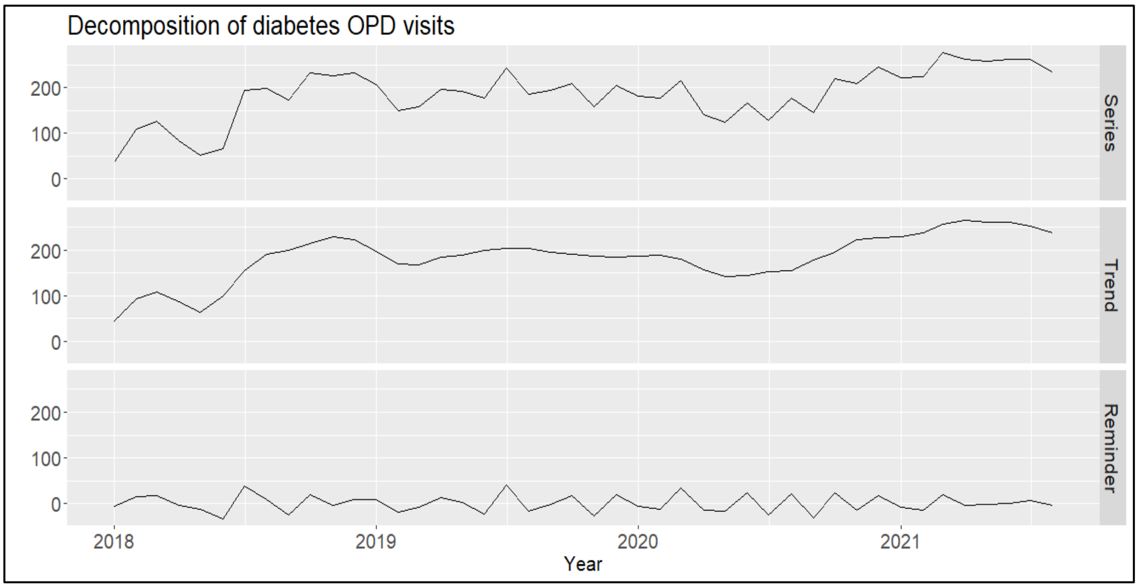

Figure S2. *Decomposition of diabetes OPD visits time series in trend component and random component*

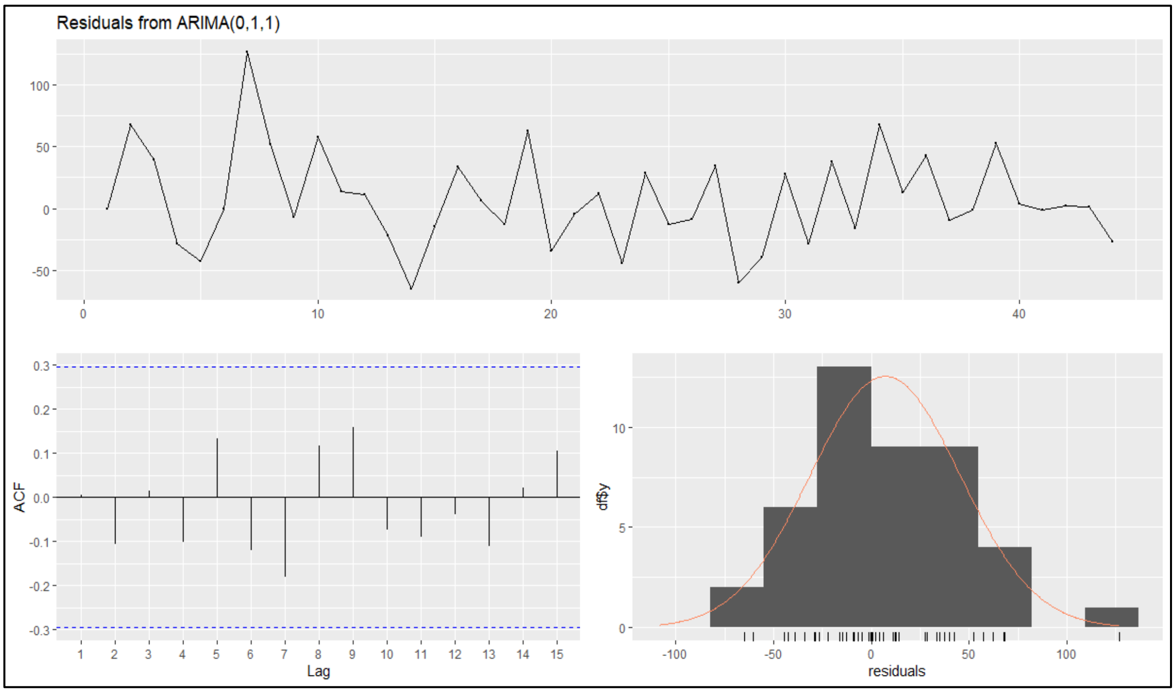

Figure S3. *Time plot of the residuals, the corresponding ACF, and a histogram from ARIMA(0,1,1) fitted on diabetes OPD visits time series (form 01/01/2018 to 31/08/2021)*

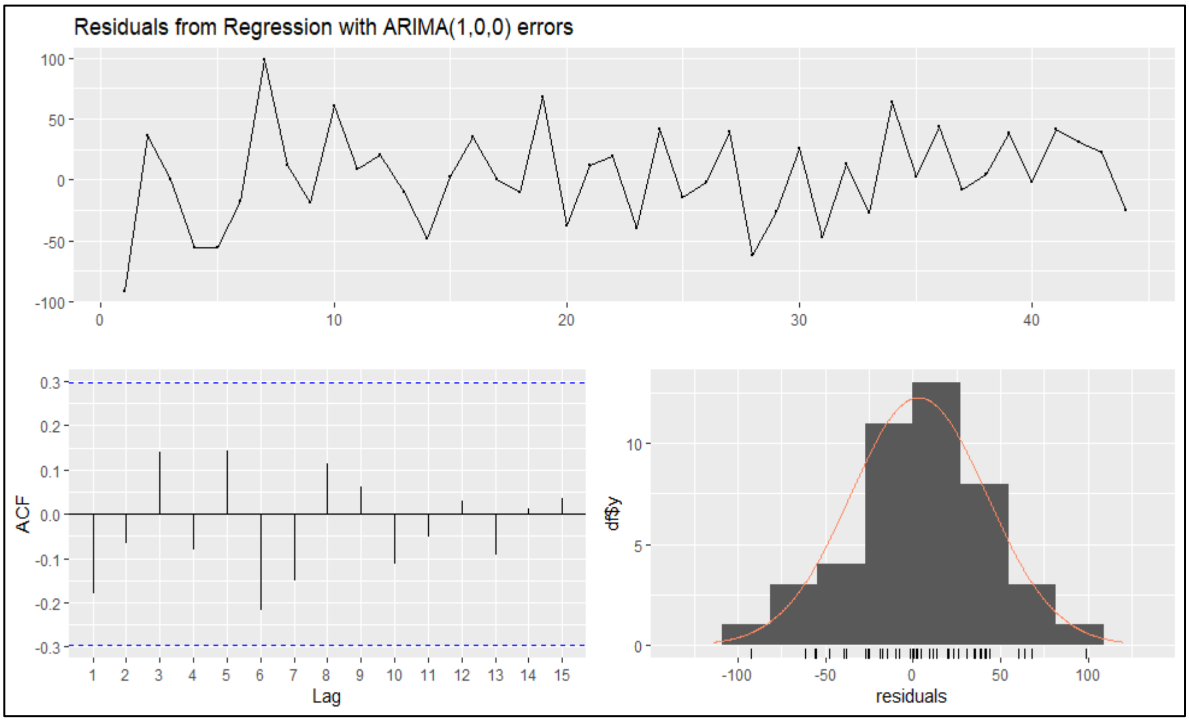

Figure S4 *Time plot of the residuals, the corresponding ACF, and a histogram from ARIMA(0,1,1) fitted on diabetes OPD visits time series using monthly number of newly registered COVID-19 cases as regressor (form 01/01/2018 to 31/08/2021).*

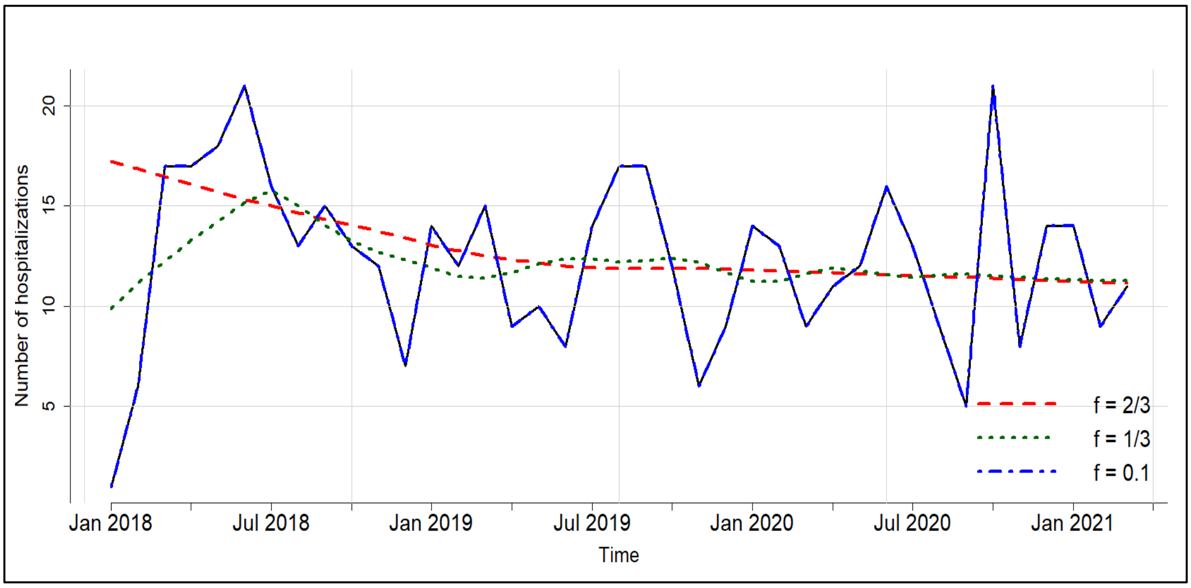

Figure S5 *Local weighted regression smoothing (LOWESS) with different smoothing parameters (f) of diabetes hospitalizations time series from January 2018 to August 2021.*

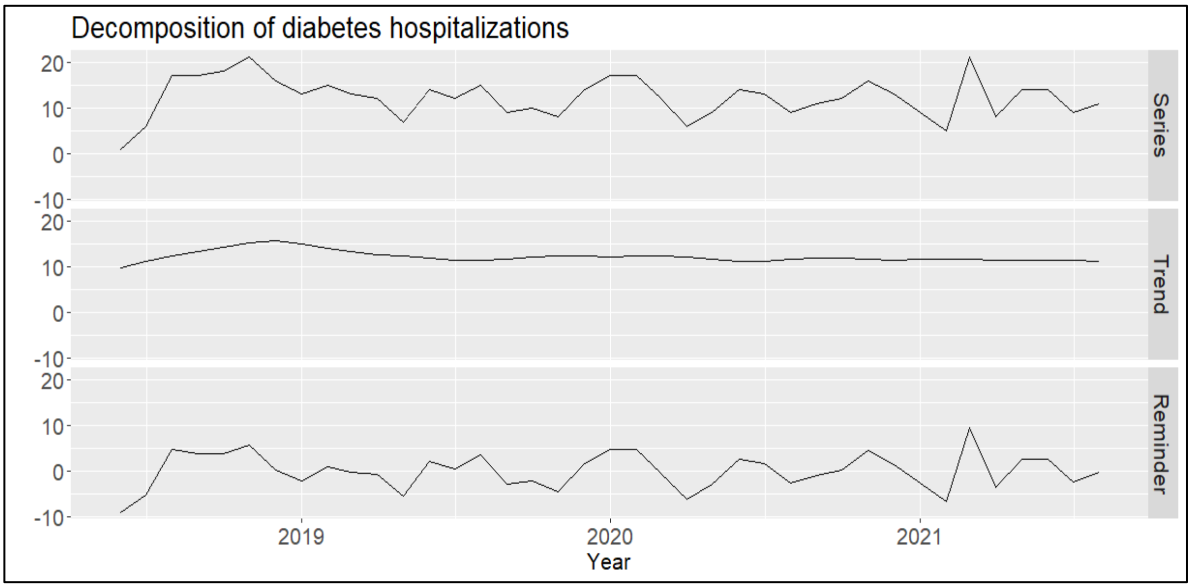

Figure S6 *Decomposition of diabetes hospitalizations time series in trend component and random component.*
